# Transfusion Thresholds and Risk Factors of Acute Kidney Injury in Gastrointestinal Oncology Surgery: Insights from a Retrospective Study

**DOI:** 10.3390/healthcare13050525

**Published:** 2025-02-28

**Authors:** Shuai Ma, Qi He, Chengcan Yang, Zhiyuan Zhou, Yining He, Chaoran Yu, Danhua Yao, Lei Zheng, Yuhua Huang, Yousheng Li

**Affiliations:** 1 Department of General Surgery, Shanghai Ninth People’s Hospital, Shanghai Jiao Tong University School of Medicine, Shanghai 200011, China; mashuai322@163.com (S.M.); heqinju@gmail.com (Q.H.); magicycc@163.com (C.Y.); wiss_zhou@hotmail.com (Z.Z.); chaoran_yu@yeah.net (C.Y.); ydh_sh9@163.com (D.Y.); blanch_zll@hotmail.com (L.Z.); huangyuhua_1991@126.com (Y.H.); 2 Biostatistics Office of Clinical Research Unit, Shanghai Ninth People’s Hospital, Shanghai Jiao Tong University School of Medicine, Shanghai 200011, China; heyining93@163.com

**Keywords:** acute kidney injury, gastrointestinal surgery, malignant tumor, postoperative complications

## Abstract

**Objectives**: To identify transfusion thresholds and risk factors for acute kidney injury (AKI) in gastrointestinal oncology surgery, enhancing early intervention and improving postoperative outcomes. **Methods**: From 2018 to 2022, 765 patients with gastric or colorectal cancer who underwent major gastrointestinal surgery were retrospectively enrolled. The primary outcome was AKI development within 7 days postoperatively. Clinicopathological characteristics and short-term outcomes were recorded and compared. **Results**: Of all enrolled patients, 39 (5.1%) developed AKI. Patients with AKI were predominantly older and had more preoperative comorbidities, lower levels of preoperative hemoglobin and serum albumin, but higher levels of blood urea nitrogen and serum creatinine (SCr). Patients developing AKI experienced higher rates of in-hospital complications (overall: 48.3% vs. 14.2%, *p* < 0.001), prolonged hospital stays (25.4 ± 22.5 days vs. 12.3 ± 7.9 days, *p* < 0.001), increased intensive care unit (ICU) admissions (53.8% vs. 22.5%, *p* < 0.001), and higher rates of 30-day re-admission (13.9% vs. 2.4%, *p* = 0.003). Significant AKI risk factors included age (per 10 years, OR: 1.567, 95% CI: 1.103–2.423, *p* = 0.043), preoperative SCr (per 10 μmol/L, OR: 1.173, 95% CI: 1.044–1.319, *p* = 0.007), intraoperative RBC transfusion (per 1000 mL, OR: 1.992, 95% CI: 1.311–3.027, *p* = 0.001 with a significant surge in AKI risk at transfusions exceeding 1500 mL), patient-controlled analgesia (protective, OR:0.338, 95% CI: 0.163–0.928, *p* = 0.033), and diuretic use (OR: 5.495, 95% CI: 1.720–17.557, *p* = 0.004). **Conclusions**: Early intervention is essential for patients with preoperative low perfusion or anemia, with particular emphasis on moderating interventions to avoid fluid overload while carefully avoiding nephrotoxic medications, thereby improving postoperative outcomes.

## 1. Introduction

Acute kidney injury (AKI) is a common and serious postoperative complication observed in about 12% of cases of all surgical procedures [1]. For abdominal surgeries specifically, the incidence rate of AKI is identified to range between 0.8% and 22.4% [2]. AKI is intricately linked to adverse clinical outcomes, which include the development of chronic kidney disease (CKD), increased postoperative complications, heightened healthcare needs, and a higher mortality rate [3,4]. The established connection between AKI and increased mortality has intensified efforts to refine risk stratification approaches [5]. However, a universally accepted method applicable either before or after surgery has yet to be developed.

Various preoperative risk factors for the development of AKI have been identified, though only a limited number are modifiable, shifting the emphasis of prevention towards procedural factors [6,7]. Among these, hemodilution, diminished oxygen delivery, intraoperative anemia, and blood transfusions are significant [8,9,10,11]. Surgical procedures could inherently lead to a certain level of ischemia–reperfusion injury to the kidneys; however, not all patients finally develop AKI, possibly due to individual differences in inflammatory reactions and the resultant renal microvascular damage [12,13]. Meanwhile, the risk of kidney injury is exacerbated by anemia and blood transfusions [14]. Nevertheless, the reported impact on AKI development is highly heterogeneous across studies, which may be attributed to the absence of standardized definitions for both the predictors and outcome, as well as the definition of AKI. Previous clinical evidence primarily derives from cardiac surgery, with scarce data available on patients with gastric or colorectal cancer, where the impact on hemodynamic changes is usually less pronounced compared to cardiac procedures. Additionally, there is a notable paucity of quantitative studies in this patient cohort.

Therefore, wo conducted this study including both preoperative and early postoperative data associated with postoperative AKI. Quantitative methods and multivariate analysis were used to identify risk factors related to patient and perioperative management associated with postoperative AKI in patients with malignant tumors who underwent gastrointestinal surgery. These findings aim to guide the development of personalized strategies for high-risk patients, focusing on optimizing transfusion thresholds and monitoring risk factors to improve clinical outcomes.

## 2. Materials and Methods

### 2.1. Study Design

This was a single-center retrospective study. Patients were enrolled between 1 January 2018 and 31 December 2022 at the Department of General Surgery, Shanghai Ninth People’s Hospital, Shanghai Jiao Tong University School of Medicine, Shanghai, China. The study protocol (SH9H-2022-T369-1) was approved by the institutional review Board of the Shanghai Ninth People’s Hospital.

### 2.2. Diagnostic Criteria

The occurrence and severity of acute kidney injury (AKI) were assessed through serum creatinine (SCr), as specified by the Kidney Disease: Improving Global Outcomes (KDIGO) definition and classification system [15]. For patients enrolled in this study, we used the latest preoperative SCr as the baseline and identified the highest SCr within 48 h and 7 days after surgery for diagnosing AKI and assessing its severity. According to the KDIGO criteria, AKI is defined by either of the following changes in SCr: an absolute increase of ≥26.5 µmol/L within 48 h or a relative increase of ≥1.5 times the baseline value within a 7-day period. AKI stages were categorized based on the changes in serum creatinine levels compared to baseline: stage 1 (SCr ≥ 26.5 µmol/L above baseline or 1.5–1.9 times the baseline value), stage 2 (an increase of 2.0–2.9 times the baseline value), or stage 3 (an increase of at least 3 times the baseline value or a postoperative serum creatinine level of ≥354 µmol/L with an increase of at least 26.5 µmol/L from baseline, or the initiation of renal replacement therapy).

### 2.3. Inclusion and Exclusion Criteria

The inclusion criteria were (1) age >18 years and (2) underwent gastrointestinal surgery for a diagnosis of gastric or colorectal cancer (confirmed by pathological examination). The exclusion criteria were as follows: (1) no major gastrointestinal surgery, (2) stage 5 CKD, (3) secondary or metastatic tumors, and (4) missing SCr data.

### 2.4. Surgical Procedure and Perioperative Management

All patients underwent either radical or palliative surgery in accordance with the latest treatment guidelines. Perioperative management was performed according to routine practice.

### 2.5. Data Collection

Patient medical records were reviewed to collect data on clinicopathological characteristics, including sex, age, preoperative height, weight and weight loss, comorbidities, smoking, alcohol use, tumor location, preoperative laboratory tests, perioperative management, complications (only events occurring before the onset of postoperative AKI were included), and short-term prognosis (length of hospital stay and 30-day re-admission). The primary outcome of our study is the occurrence of postoperative AKI.

### 2.6. Statistical Analysis

The data were presented as mean ± standard deviation. Normally distributed data were compared between groups using the two-sample independent Student’s *t*-test. Non-normally distributed data were compared using the Mann–Whitney U test. Categorical variables were expressed as counts (%), and group comparisons were conducted using the *χ*^2^ test or Fisher’s exact test. Univariate and multivariate analyses using binary logistic regression were performed to identify risk factors for postoperative AKI. A generalized additive model (GAM) was utilized to evaluate dose–effect relationships. Variables with a significance level of *p* < 0.05 in the univariate analysis were included in the multivariate analysis. Results were considered statistically significant at a *p* < 0.05. Statistical analyses were conducted using IBM SPSS Statistics software (version 25 for Mac, IBM Corp., Armonk, NY, USA) and R software (version 4.3.0 for Mac).

## 3. Results

### 3.1. Baseline Clinicopathological Characteristics

**Figure 1:**
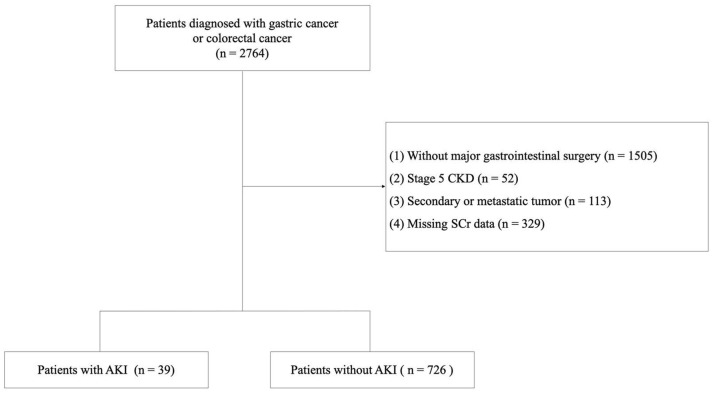
Flowchart illustrating the study approach. CKD, chronic kidney disease; SCr, serum creatinine; AKI, acute kidney injury.

As shown in Figure 1 and Table 1, this study enrolled 765 patients admitted between January 2018 and December 2022. Thirty-nine (5.1%) patients experienced postoperative AKI, which was dominated by KDIGO stage 1 (3.7%), followed by stages 2 (1.2%) and 3 (0.3%). Among all patients, 8 (3.3%) with gastric cancer and 31 (6.0%) with colorectal cancer presented postoperative AKI. All patients were diagnosed with gastric or colorectal cancer and underwent major gastrointestinal surgery. Among all enrolled cases (Table 1), patients in the AKI group were significantly older than patients in the non-AKI group (75.2 ± 11.8 vs. 64.8 ± 11.8 years, *p* < 0.001) and were preoperatively diagnosed with more cerebrovascular (20.5% vs. 7.7%, *p* = 0.012) and cardiovascular (23.1% vs. 10.7%, *p* = 0.033) comorbidities. Laboratory test results showed that the AKI group had lower hemoglobin (Hb) (109.9 ± 23.0 vs. 119.9 ± 21.5 g/L, *p* = 0.003) and albumin (Alb) (37.0 ± 3.8 vs. 39.5 ± 4.4 g/L, *p* < 0.001) baseline levels, with higher blood urea nitrogen (BUN) (6.7 ± 3.4 vs. 5.4 ± 2.1 mmol/L, *p* = 0.021) and SCr levels (95.7 ± 45.0 vs. 68.8 ± 20.7 mol/L, *p* < 0.001). Patients with AKI received a higher volume of intraoperative red blood cell (RBC) transfusion (374.4 ± 472.8 mL vs. 180.7 ± 375.0 mL, *p* = 0.001), postoperative RBC transfusion (1394.9 ± 2293.6 mL vs. 136.0 ± 460.2 mL, *p* < 0.001), and a higher dose of albumin intravenous infusion (236.6 ± 373.7 g vs. 51.9 ± 90.0 g, *p* < 0.001). A higher proportion of diuretic (89.7% vs. 34.0%, *p* < 0.001) and vasopressor (89.7% vs. 69.4%, *p* = 0.007) use and a lower proportion of patient-controlled analgesia (PCA) (43.6% vs. 76.4%, *p* < 0.001) were observed in patients with AKI.

### 3.2. Short-Term Outcomes

The AKI group had unfavorable short-term outcomes compared to the non-AKI group (Table 2), with a prolonged duration of postoperative hospital stay (25.4 ± 22.5 vs. 12.3 ± 7.9 d, *p* < 0.001), a higher rate of intensive care unit (ICU) admission (53.8% vs. 22.5%, *p* < 0.001), a longer length of stay in the ICU (once admitted) (10.5 ± 11.2 vs. 4.3 ± 3.9, *p* = 0.002), a higher rate of requirement for ventilatory support (46.2% vs. 6.7%, *p* < 0.001), and a higher rate of renal replacement therapy (RRT) (5.1% vs. 0.1%, *p* = 0.007). The AKI group had more postoperative complications (48.3% vs. 14.2%, *p* < 0.001), with a greater proportion of infections (25.6% vs. 8.1%, *p* < 0.001) and hemorrhage (12.8% vs. 2.3%, *p* = 0.004). Moreover, the AKI group had a higher 30-day re-admission (13.9% vs. 2.4%, *p* = 0.003).

### 3.3. Risk Factors for Postoperative AKI

Univariate and multivariate analyses were performed to explore the risk factors for postoperative AKI (Table 3). In the univariate analysis, age, preoperative cerebrovascular comorbidities, preoperative cardiovascular comorbidities, intraoperative RBC transfusion, postoperative RBC transfusion, postoperative colloid infusion, albumin infusion, PCA, diuretics, vasopressor use, postoperative complications of infection, obstruction, and hemorrhage, and preoperative laboratory tests for Hb, Alb, BUN, SCr, and glucose (Glu) were identified as risk factors. In the subsequent multivariate analysis, age (per 10 years, OR: 1.567, 95% CI: 1.103–2.423, *p* = 0.043), intraoperative RBC transfusion (per 1000 mL, OR: 1.992, 95% CI: 1.311–3.027, *p* = 0.001), diuretics (OR: 5.495, 95% CI: 1.720–17.557, *p* = 0.004), and preoperative laboratory SCr levels (per 10 μmol/L, OR: 1.173, 95% CI: 1.044–1.319, *p* = 0.007) were identified as independent risk factors for postoperative AKI. PCA (OR: 0.388, 95% CI: 0.163–0.928, *p* = 0.033) was identified as an independent protective factor.

### 3.4. Dose–Effect Relationship Between Intraoperative RBC Transfusion

**Figure 2:**
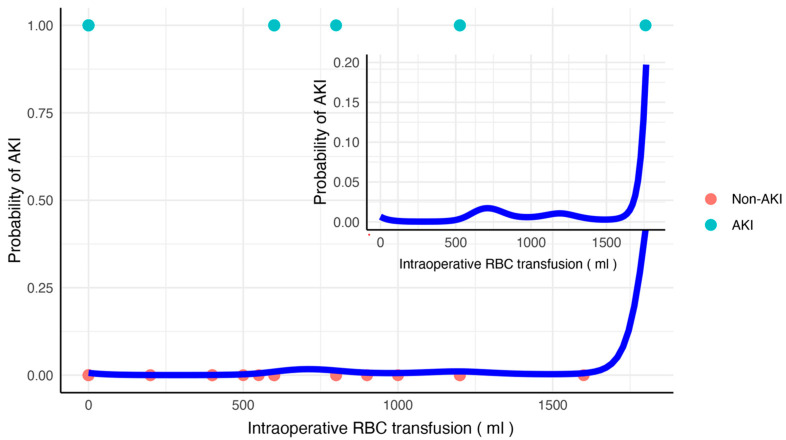
Dose–effect relationship between intraoperative red blood cell transfusion. The dose–effect relationship between intraoperative RBC transfusion and probability was assessed using a generalized additive model with other independent multivariate risk factors. Red dots indicate cases without postoperative AKI. Green dots indicate postoperative AKI. The blue lines represent the modeled data. RBC, red blood cell; AKI, acute kidney injury.

As shown in Figure 2, GAM was used to assess the non-linear dose–effect relationship between intraoperative RBC transfusion and postoperative AKI. When the volume of intraoperative RBC transfusion volume was <1500 mL, the probability of AKI was maintained at a level close to zero. When it exceeded 1500 mL, the probability of AKI surged to increase.

## 4. Discussion

AKI is recognized as one of the most common complications of major abdominal surgery, with the second-highest incidence of AKI occurring after cardiac surgery [16]. A strong association between postoperative AKI and adverse outcomes when incorporated with the degree of kidney stress or injury has been described [17]. Our study suggested that an incidence of AKI of 5.1% among patients with malignant tumors who underwent gastrointestinal surgery resulted in obvious detrimental short-term surgical outcomes despite the majority of cases of AKI being at KDIGO stage 1. Given the substantial prevalence of AKI and its unfavorable consequences, it is crucial to prioritize AKI prevention and the alleviation of additional damage once early-stage AKI evidence is present or predicted. Therefore, this study aimed to identify the risk factors to guide the perioperative assessment and the preventive therapies to achieve these goals. In this study, we found that older age, intraoperative transfusion of RBC, diuretics, and preoperative laboratory SCr levels were independent risk factors for postoperative AKI, while PCA was shown as an independent protective factor.

Preoperative conditions such as anemia, dehydration, and low-perfusion states play a pivotal role in influencing intraoperative and postoperative AKI factors [18]. Our study showed that intraoperative RBC transfusion was an independent risk factor for postoperative AKI. This factor adds a 1.992-fold greater risk of developing AKI when each additional 1000 mL intraoperative RBC transfusion is administered. Two European institutes [19] conducted a retrospective study, suggesting that RBC transfusion was an independent risk factor for AKI, increasing the risk in a dose-dependent manner, which is in accordance with our results. There are three possible explanations for this. First, a massive volume of intraoperative RBC transfusion often arises from severe preoperative low-perfusion conditions [18], which can be caused by a malignant tumor or intraoperative bleeding. The fundamental concepts commonly understood by most medical professionals with respect to AKI include acute tubular necrosis and prerenal azotemia [20]. Acute tubular necrosis, a manifestation of intrinsic AKI, occurs due to prolonged and severe renal hypoperfusion, illustrating the potential for low-perfusion states to precipitate AKI [20]. Second, a high volume of intraoperative RBC transfusion is usually required in cases of severe anemia. A meta-analysis [21] showed that perioperative anemia was associated with increased rates of AKI, stroke, infection, and 30-day mortality. Preoperative anemia has been reported as a risk factor for postoperative AKI [22], and anemia in ICU patients is a known risk factor for long-term mortality [23]. A Chinese three-center study [24] reported that maintaining Hb levels > 90 g/L by RBC transfusion was associated with increased incidence of AKI. The relationship between anemia and AKI can be explained by ischemia–reperfusion injury. Finally, the massive volume of intraoperative RBC transfusions led to fluid overload, which in turn led to AKI [25]. Findings from a meta-analysis [25] indicated correlations between AKI and both fluid overload and cumulative fluid balance. The researchers elaborated on how fluid overload could impair cardiac function, elevate intra-abdominal pressure, and lead to renal venous congestion. Therefore, preoperative optimization of the patient’s general condition is crucial for reducing the risk of postoperative AKI, particularly in patients presenting with dehydration or anemia due to chronic or acute blood loss. These conditions can compromise renal perfusion even before surgery. Furthermore, the use of contrast media for imaging [26] in patients with impaired renal function should be carefully evaluated to minimize contrast-induced nephropathy. Early correction of these factors with appropriate hydration would be important. A feasible way to prevent AKI while avoiding a massive volume of postoperative transfusion of RBC is to correct low-perfusion conditions and anemia step by step preoperatively, if possible. When necessary, leukocyte-depleted transfusions [27] help improve the patient’s condition prior to surgery and potentially reduce AKI risk.

Another risk factor in the preoperative phase we found was that preoperative SCr levels were associated with an increased risk of AKI despite excluding patients with stage 5 CKD. At baseline, the AKI group had lower levels of hemoglobin and albumin and higher levels of BUN and SCr, although these laboratory test results were within the normal range. Lower levels of Hb and Alb often suggest low perfusion conditions that may cause prerenal azotemia. An essential strategy to prevent AKI involves addressing and managing underlying causes or trigger factors [17]. When prerenal factors are deemed contributory, prompt identification is essential, followed by immediate initiation of hemodynamic resuscitation and the maintenance or rapid restoration of intravascular volume [20,28]. For numerous patients, the placement of a peripheral intravenous catheter and expeditious intravenous fluid administration suffice to accomplish this procedure. Preoperative neoadjuvant chemotherapy is theoretically another factor [29] in the preoperative phase that could influence the risk of postoperative AKI. However, due to the retrospective nature of our study, detailed information regarding preoperative chemotherapy regimens and their specifics was not consistently available during data collection. This limitation highlights one of the reasons we included preoperative serum creatinine as a variable, as it can, to some extent, provide indirect insights into the patient’s preoperative condition and potential renal impacts. Furthermore, we acknowledge that other preoperative advanced CKD stages could also compromise renal function and potentially influence our findings. Still, the retrospective design made it difficult to accurately identify and exclude all such cases, which is why we primarily relied on preoperative SCr to reflect a range of renal function statuses.

In addition, we identified older age as an independent risk factor for postoperative AKI, consistent with previous studies [30]. Advanced age may contribute to AKI through mechanisms such as reduced baseline renal function, the presence of comorbidities (e.g., diuretic use, heart failure, and chronic low perfusion), and increased vulnerability to hemodynamic changes [30]. However, the potential collinearity between age and these variables poses challenges in disentangling their individual effects. Given the limited sample size of our single-center study, further subgroup analyses were not feasible to elucidate these complex relationships.

For the intraoperative phase, we aimed to explore the impact of surgical procedures on postoperative AKI. Previous literature [31] reported that lymphadenectomy, depending on its range, can theoretically result in significant serum loss, which may exacerbate renal function by imposing additional stress on the renal clearance system. Likewise, the type, duration, and technique of surgery [32] may influence AKI risk through similar mechanisms. Unfortunately, in our study, we did not observe significant differences in postoperative AKI incidence between patients with gastric and colorectal cancers. This is likely due to the limited number of AKI cases in our cohort. Specifically, the AKI group comprised 8, 18, and 13 cases with gastric, colon, and rectal cancers, respectively (Table 1). Furthermore, the variety of surgical procedures and lymphadenectomy types inherent to gastrointestinal oncology makes it challenging to conduct more detailed subgroup analyses in this single-center study.

During the intraoperative and postoperative period, meticulous fluid management is essential to maintain hemodynamic stability and renal perfusion, especially in elderly patients or those with pre-existing comorbidities [32,33]. Maintaining a balanced fluid input and output during surgery can help prevent fluid overload and its adverse effects on kidney function. Goal-directed therapy could be a feasible way to improve renal perfusion and oxygenation in high-risk patients undergoing gastrointestinal surgery for malignant tumors. However, under conditions of severe anemia or massive hemorrhage, it is difficult to balance low perfusion and avoid fluid overload. In our study, we found that the risk of developing AKI was relatively low with non-massive RBC transfusions. The probability of postoperative AKI increased until the volume of RBC transfusions reached >1500 mL. Therefore, to correct preoperative anemia, it is reasonable to assume that multiple minor preoperative RBC transfusions may benefit these patients more than massive transfusions at one time. However, a prior study [34] presented disputed conclusions, indicating that even 1–2 RBC units increased the risk of major adverse events, including AKI. It is noteworthy that their studies focused on the population undergoing coronary artery bypass grafting surgery rather than abdominal surgery, and the speed of transfusion has not been mentioned. The possible reasons may be attributed to the more critical preoperative status compared with patients with gastric or colorectal cancer. To our knowledge, there is no consensus on the optimal transfusion speed to balance low-perfusion conditions and avoid fluid overload. A rat experiment [35] suggested that transfusion volume dose-dependently increased left ventricular end-diastolic pressure (LVEDP), with the speed of transfusion rapidly elevating LVEDP at higher transfusion volumes, potentially underlying AKI. However, this assumption needs to be confirmed in further clinical trials.

Other intraoperative and postoperative risk factors for the development of postoperative AKI included the use of nephrotoxic drugs or agents, which, in our study, comprised PCA and diuretics. It has been documented [36] that the choice of drugs should consider their nephrotoxic potential in addition to providing the desired pharmacological effects. Our study demonstrated that PCA serves as a protective factor against AKI. PCA is a method employed for delivering analgesics to address acute postoperative pain [37]. Within our center, PCA predominantly involves the administration of opioids like fentanyl. Non-steroidal anti-inflammatory drugs (NSAIDs) represent viable alternative analgesics for managing moderate to severe pain [38] by potentially diminishing the need for postoperative opioids [39]. However, there is concern that NSAIDs may induce AKI owing to nephrotoxicity [40]. A study [41] compared ketorolac-based and fentanyl-based PCA and found that ketorolac-based PCA was independently associated with the development of postoperative AKI. Another study [42] suggested a controversial conclusion that no association was identified between postoperative AKI and the use of NSAIDs or opioids for PCA. However, their study [42] involved nephrectomy, which may have had a potential effect on AKI. Our study found that diuretic use was one risk factor for AKI. Unlike the nephrotoxicity of NSAIDs, the effect of diuretics, such as furosemide, on AKI was observed in two ways. First, furosemide can acidify the urine, and acidic urine may result in the formation of methemoglobin casts in patients with severe intravascular hemolysis [43], which are potentially nephrotoxic and can cause further renal failure. However, on the other hand, furosemide could also be used in the setting of AKI caused by circulatory overload [44]. Opting for a conservative fluid management strategy can lead to positive outcomes by incorporating a higher utilization of loop diuretics in critically ill patients with the aim of avoiding positive fluid balance. A quantitative study [44] revealed that equivalent doses of up to 80 mg/day of furosemide did not have a significant association with mortality. Our study suggests that diuretic use increases the risk of AKI. Furosemide is a common diuretic used in our center, and AKI may be attributed to its nephrotoxicity. However, we did not calculate the dose–effect relationship or clarify the effects of other diuretic categories further.

Our study is subject to several limitations. First, this was a retrospective study conducted at a single center. Owing to the low incidence of AKI, only a small sample of patients with AKI was included, although the total number of patients with malignant tumors who underwent gastrointestinal surgery was considerable. Second, we did not use any quasi-experimental method to construct an artificial control group by matching each treated unit with a nontreated unit of similar characteristics because the number of cases would be lost if matching was performed. Third, we did not compare the differences between gastric cancer patients and colorectal cancer patients because of the small sample size of gastric cancer patients. Additionally, broadening the inclusion criteria created a more comprehensive but less homogeneous cohort. We also included numerous covariates in the baseline table, some of which may not necessarily be primary risk factors, but their imbalance could serve as confounding factors that influence the observed outcomes, leading to potential research bias. Although our multivariate analysis can partially address this issue by identifying potential independent risk factors, the small number of AKI cases limits the power to fully adjust for all confounders. Some variables may still have potential multicollinearity, and further clinical studies with larger sample sizes are needed to clarify these relationships.

## 5. Conclusions

Our study underscores the importance of identifying intraoperative blood transfusion thresholds and recognizing factors that forecast the risk of acute kidney injury following gastrointestinal cancer surgeries. Proactive measures, such as tailoring interventions to manage low perfusion, anemia, and fluid overload, are critical for enhancing patient recovery and surgical outcomes. These findings highlight the need for personalized preventative strategies, focusing on optimizing transfusion thresholds and enhancing the monitoring of high-risk patients to mitigate postoperative AKI and improve clinical outcomes.

## Figures and Tables

**Table 1:** Characteristics of patients with and without AKI.

	AKI (n = 39)	Non-AKI (n = 726)	*p*
Sex (Male, %)	26 (66.7%)	457 (62.9%)	0.639
Age (years)	75.2 ± 11.8	64.8 ± 11.8	**<0.001**
Height (cm)	162.7 ± 9.4	164.1 ± 7.7	0.511
Weight (kg)	67.8 ± 15.5	63.7 ± 10.7	0.114
Weight loss (kg)	0.8 ± 1.8	1.1 ± 2.9	0.459
Comorbidity (%)	29 (74.4%)	394 (54.3%)	**0.014**
Diabetes (%)	9 (23.1%)	117 (16.1%)	0.254
Hypertension (%)	19 (48.7%)	271 (37.3%)	0.153
Cerebrovascular (%)	8 (20.5%)	56 (7.7%)	**0.012**
Cardiovascular (%)	9 (23.1%)	78 (10.7%)	**0.033**
Smoking (%)	3 (7.7%)	94 (13.0%)	0.461
Alcohol use (%)	1 (2.6%)	62 (8.6%)	0.242
Site			
Gastric/colon/rectum	8/18/13	238/254/234	0.218
Preoperative laboratory tests			
WBC (×10^9^/L)	7.0 ± 2.2	6.4 ± 2.3	0.110 *
Hb (g/L)	109.9 ± 23.0	119.9 ± 21.5	**0.003** *
Alb (g/L)	37.0 ± 3.8	39.5 ± 4.4	**<0.001** *
Alt (U/L)	14.5 ± 7.4	19.0 ± 16.9	0.055 *
BUN (mmol/L)	6.7 ± 3.4	5.4 ± 2.1	**0.021** *
SCr (μmol/L)	95.7 ± 45.0	68.8 ± 20.7	**<0.001** *
Glu (mmol/L)	6.2 ± 2.2	5.6 ± 1.6	0.250 *
Minimal invasive surgery (%)	18 (46.2%)	432 (59.5%)	0.113
Intraoperative RBC transfusion (mL)	374.4 ± 472.8	180.7 ± 375.0	0.001 *
Intraoperative colloid (mL)	243.6 ± 378.2	247.9 ± 307.1	0.654 *
Postoperative RBC transfusion (mL)	1394.9 ± 2293.6	136.0 ± 460.2	<0.001 *
Postoperative colloid (mL)	65.8 ± 171.3	32.4 ± 180.0	0.016 *
Albumin intravenous infusion (mL)	236.6 ± 373.7	51.9 ± 90.0	<0.001 *
PCA (%)	17 (43.6%)	554 (76.4%)	**<0.001**
Diuretics (%)	35 (89.7%)	247 (34.0%)	**<0.001**
Vasopressor (%)	35 (89.7%)	504 (69.4%)	**0.007**
Aminoglycoside antibiotics (%)	7 (17.9%)	182 (25.1%)	0.315
Non-selective COX-2 inhibitors (%)	36 (92.3%)	657 (90.5%)	0.706
Selective COX-2 inhibitors (%)	10 (25.6%)	133 (18.3%)	0.253

AKI, acute kidney injury; WBC, white blood cell; Hb, hemoglobin; Alb, albumin; Alt, alanine aminotransferase; BUN, blood urea nitrogen; SCr, serum creatinine; Glu, glucose; RBC, red blood cell; PCA, patient-controlled analgesia; COX-2, cyclooxygenase-2. Quantitative data were compared using the *t*-test, with * indicating the use of the Mann-Whitney U test. Statistically significant values are indicated in bold.

**Table 2:** Short-term prognosis of patients with and without AKI.

	AKI (n = 39)	Non-AKI (n = 726)	*p*
Postoperative LOS (d)	25.4 ± 22.5	12.3 ± 7.9	**<0.001**
First oral feeding (d)	8.8 ± 12.4	5.3 ± 4.4	0.255
ICU stay (d)	21 (53.8%)	163 (22.5%)	**<0.001**
Length of ICU stay (d)	10.5 ± 11.2	4.3 ± 3.9	**0.002**
Ventilator (%)	18 (46.2%)	49 (6.7%)	**<0.001**
RRT (%)	2 (5.1%)	1 (0.1%)	**0.007**
Complications (%)	19 (48.3%)	103 (14.2%)	**<0.001**
Infection (%)	10 (25.6%)	59 (8.1%)	**<0.001**
Leakage (%)	3 (7.7%)	16 (2.2%)	0.067
Obstruction (%)	1 (2.6%)	13 (1.8%)	0.522
Hemorrhage (%)	5 (12.8%)	17 (2.3%)	**0.004**
30 d re-admission (%)	5 (13.9%)	16 (2.4%)	**0.003**

AKI, acute kidney injury; LOS, length of hospital stay; ICU, intensive care unit; RRT, renal replacement therapy. Statistically significant values are indicated in bold.

**Table 3:** Risk factors of postoperative AKI after major gastrointestinal surgery in malignant patients.

	Univariate			Multivariate		
	OR	95% CI	*p*	OR	95% CI	*p*
Sex						
Female	Ref					
Male	1.177	0.595–2.330	0.639			
Age (per 10 years)	2.501	1.767–3.540	**<0.001**	1.567	1.103–2.423	**0.043**
Weight loss	0.940	0.800–1.106	0.458			
Comorbidity						
Diabetes	1.562	0.722–3.375	0.257			
Hypertension	1.595	0.836–3.042	0.156			
Cerebrovascular	3.088	1.355–7.036	**0.007**			
Cardiovascular	2.492	1.141–5.443	**0.022**			
Site						
Gastric	Ref					
Colon	2.108	0.900–4.939	0.086			
Rectum	1.653	0.673–4.061	0.273			
Minimal invasive surgery	0.531	0.276–1.023	0.059			
Intraoperative RBC transfusion (per 1000 mL)	2.331	1.296–4.195	**0.005**	1.992	1.311–3.027	**0.001**
Intraoperative colloid (per 1000 mL)	2.176	0.919–5.149	0.077			
Postoperative RBC transfusion (per 1000 mL)	2.938	2.088–4.134	**<0.001**			
Postoperative colloid (per 1000 mL)	1.561	1.249–1.949	**<0.001**			
Albumin intravenous infusion (per 100 g)	1.781	1.457–2.178	**<0.001**			
PCA	0.239	0.124–0.460	**<0.001**	0.388	0.163–0.928	**0.033**
Diuretics	16.969	5.963–48.287	**<0.001**	5.495	1.720–17.557	**0.004**
Vasopressor	3.854	1.354–10.975	**0.012**			
Aminoglycoside antibiotics	0.654	0.284–1.507	0.319			
Non-selective COX-2 inhibitors	1.260	0.378–4.199	0.706			
Selective COX-2 inhibitors	1.537	0.731–3.232	0.256			
Complications						
Infection	3.898	1.811–8.390	**0.001**			
Leakage	3.698	1.030–13.270	**0.045**			
Obstruction	1.443	0.184–11.324	0.727			
Hemorrhage	6.133	2.136–17.611	**0.001**			
Preoperative laboratory tests						
WBC (per 10 × 10^9^/L)	2.363	0.686–8.146	0.173			
Hb (per 10 g/L)	0.809	0.697–0.940	**0.006**			
Alb (per 10 g/L)	0.272	0.123–0.600	**0.001**			
Alt (per 10 U/L)	0.679	0.433–1.065	0.092			
BUN (per 10 mmol/L)	4.640	1.650–1.466	**<0.001**			
SCr (per 10 μmol/L)	1.325	1.197–1.466	**<0.001**	1.173	1.044–1.319	**0.007**
Glu (per 5 mmol/L)	2.226	1.049–4.721	**0.037**			

AKI, acute kidney injury; RBC, red blood cell; PCA, patient-controlled analgesia; COX-2, cyclooxygenase-2; WBC, white blood cell; Hb, hemoglobin; Alb, albumin; Alt, alanine aminotransferase; BUN, blood urea nitrogen; SCr, serum creatinine; Glu, glucose. Statistically significant values are indicated in bold.

## Data Availability

The datasets are available from the corresponding author upon reasonable request.
